# Microbial Community Dynamics in Marine Water: Influence of Hydrocarbon Type and Exposure Time

**DOI:** 10.1002/tox.70070

**Published:** 2026-03-06

**Authors:** Karen C. F. Santaren, Douglas A. Monteiro, Eliene S. Lopes, Diogo A. Jurelevicius, Lucy Seldin

**Affiliations:** ^1^ Departamento de Microbiologia Geral Instituto de Microbiologia Paulo de Góes, Universidade Federal do Rio de Janeiro (UFRJ) Rio de Janeiro (RJ) Brazil

**Keywords:** bioremediation, hydrocarbon degradation over time, hydrocarbon mixtures, hydrocarbon‐degrading bacteria, marine water contamination, microbial community succession

## Abstract

Marine environments are frequently impacted by petroleum‐derived hydrocarbons, which pose ecological risks because of their toxic, mutagenic, and carcinogenic potential. Microorganisms play a crucial role in hydrocarbon degradation, and understanding the influence of time and hydrocarbon type on microbial dynamics helps improve bioremediation strategies. In this study, marine water samples were used to assemble microcosms containing different hydrocarbon contaminants (naphthalene–NAF, crude oil–OL, and a synthetic mixture–MIX) and were incubated for 7, 15, 30, 45, and 60 days under controlled conditions. The bacterial community structure and composition were analyzed via 16S rRNA gene sequencing combined with functional prediction via PICRUSt2. Alpha diversity differed significantly among contaminants, with OL and MIX supporting more diverse communities. Beta diversity was shaped by both contaminant type and incubation time. Hydrocarbon‐degrading taxa such as *Marinobacter*, *Alteromonas*, *Pseudomonas*, and *Thalassospira* dominated at different stages, which was consistent with a functional progression from aliphatic oxidation (K00496) to successive steps of aromatic ring degradation (K00529, K00448, K07104). Together, these results demonstrate that hydrocarbon type and exposure time shape microbial succession, driving taxonomic differentiation and metabolic specialization, and providing insights for improving bioremediation strategies in marine environments.

## 1 Introduction

The contamination of oceans by hydrocarbons, particularly polycyclic aromatic hydrocarbons (PAHs), poses significant risks to both marine biota and human health through direct contact, inhalation, or ingestion. Exposure to these compounds has been associated with carcinogenicity, mutagenicity, and toxicity and often results in mortality [1, 2].

Bacteria play a key role in the biodegradation of petroleum‐derived pollutants by converting complex organic compounds into inorganic constituents and biomass. This process contributes to contaminant mineralization and provides natural attenuation of hydrocarbons in the environment, supporting bioremediation approaches. Such bioremediation‐based strategies are crucial for mitigating the impacts of oil spills and chronic hydrocarbon contamination in marine ecosystems [3, 4].

In petroleum‐contaminated environments, hydrocarbon‐degrading microorganisms tend to become significantly more abundant than in noncontaminated environments. The presence of the pollutant acts as a selective pressure, inhibiting sensitive microbial groups while promoting the rapid proliferation of degraders, which consequently become dominant within the community [4, 5, 6]. Moreover, other community members not directly involved in hydrocarbon breakdown contribute to essential ecological functions, such as nutrient exchange, cometabolism, and biosurfactant production that support and increase the activity of primary degraders [6].

The degradation of different types of hydrocarbons—aliphatic (no aromatic rings in the structure), monocyclic aromatic (one aromatic ring in the structure), and polycyclic aromatic (more than one aromatic ring in the structure)—depends on the specific metabolic capabilities of distinct bacterial groups. In general, alkanes (C_*n*_H_2*n*+2_) tend to degrade more rapidly, whereas hydrocarbons with more complex structures are more persistent in the environment. This is partly due to an increased number of aromatic rings and the structural complexity of the molecules, which confer greater electrochemical stability and hydrophobicity, thereby hindering enzymatic access and consequently microbial degradation [7, 8, 9].

Therefore, this process gives rise to microbial ecological succession, initially characterized by the emergence of organisms that rapidly respond to more readily available substrates, such as alkanes. For example, early stages show an increase in alkane‐degrading taxa such as *Alcanivorax* and *Arcobacter* [10]. Over time, as these simpler compounds are consumed, there is a progressive enrichment of more specialized taxa capable of degrading recalcitrant compounds, including complex PAHs. For example, *Cycloclasticus*, *Colwellia*, and *Pseudoalteromonas* are commonly present in the late stages of contamination, as reported in oil spill incidents [11].

This study investigated the taxonomic and successional dynamics of seawater microbial communities exposed to three hydrocarbon sources—naphthalene, crude oil, and a defined mixture of aliphatic, monocyclic, and polycyclic aromatic compounds—over a 60‐day period. Previous microcosm studies have often been limited to single compounds or short‐term assessments, which may not fully represent the environmental complexity of hydrocarbon contamination.

The hypotheses of this study are that contaminant complexity drives distinct successional trajectories. Simpler compounds are expected to favor early responders, whereas complex mixtures will enrich specialized degraders. Understanding these dynamics is critical because hydrocarbon pollutants are both diverse and persistent in marine environments. Gaining such knowledge will help clarify the mechanisms of natural attenuation and support the development of effective bioremediation strategies.

## 2 Methods

### 2.1 Marine Water Sampling

Five liters of marine water were sampled in Grumari Beach (Barra da Tijuca, Rio de Janeiro, Brazil, 23°02′52.7″ S 43°31′17.6″ W) in March 2022 at a distance of 1 m into the sea and up to 30 cm below the water surface. The water temperature (26°C) was determined with a conventional thermometer at the time of sampling. The water pH (7) and salinity rate (3.4%) were measured via a portable SevenGo instrument (Mettler Toledo‐Inlab, Leicester, UK). The samples were stored at room temperature until the next day and then used for bacterial enrichment through microcosm assembly.

### 2.2 Microcosm Assembly

Microcosms were assembled by using 25 mL of seawater and the following hydrocarbon contaminants (in triplicate): 1% naphthalene (NAF), a model PAH; a microdroplet of crude oil (OL); and a controlled mixture of different types of hydrocarbons (aliphatic—hexane, hexadecane, and triacontane; monocyclic aromatic—benzene and toluene; and polycyclic aromatic—naphthalene, phenanthrene, and anthracene—0.125% each, totaling 1% [MIX]). The solid hydrocarbons (TR, NAF, FE, and AN) were weighed on an analytical balance and directly added to the flasks, whereas the liquid hydrocarbons (HA, HD, BE, and TO) were measured in appropriate volumes and pipetted directly into the microcosms. Crude oil was added as a microdroplet using a pipette because of its high viscosity. The microcosms were incubated at room temperature (24°C–28°C) under agitation for five different durations: 7, 15, 30, 45, and 60 days. A control (TZ) was also added in triplicate, containing 25 mL of seawater, which was frozen immediately after the sampling (Time 0). In total, the experiment consisted of 48 samples: 3 contaminants × 3 replicates × 5 time points (45 samples), plus 3 control samples (TZ) (Table 1).

**TABLE 1:** Description of contaminants used for marine water contamination and hydrocarbon‐degrading bacteria enrichment over various incubation times (7, 15, 30, 45, and 60 days).

Code	Contaminants	Chemical formula of contaminants	Manufacturer	Hydrocarbon's type
TZ	Marine water frozen right after sampling	No contaminants (control)	—	—
NAF	Naphthalene	C_10_H_8_	Sigma–Aldrich, St. Louis, USA	Polycyclic aromatic
MIX	Hexane	C_6_H_14_	Merck, Darmstadt, Germany	Aliphatic
	Hexadecane	CH_3_(CH_2_)_14_CH_3_	Sigma–Aldrich, St. Louis, USA	Aliphatic
	Triacontane	C_30_H_62_	Sigma–Aldrich, St. Louis, USA	Aliphatic
	Benzene	C_6_H_6_	Sigma–Aldrich, St. Louis, USA	Monocyclic aromatic
	Toluene	C_7_H_8_	Reagen, Ringoes, USA	Monocyclic aromatic
	Naphthalene	C_10_H_8_	Sigma–Aldrich, St. Louis, USA	Polycyclic aromatic
	Anthracene	C_14_H_10_	Sigma–Aldrich, St. Louis, USA	Polycyclic aromatic
	Phenanthrene	C_14_H_10_	Sigma–Aldrich, St. Louis, USA	Polycyclic aromatic
OL	Crude oil	—	—	Natural mixture

### 2.3 DNA Extraction for Bacterial Community Assessment

After the defined incubation periods, the contents of the microcosms were frozen for subsequent DNA extraction. Water samples were filtered through 0.22 μm Millipore membranes, followed by DNA extraction via the Fast DNA Spin Kit for Soil (MP Biomedicals, California, USA) according to the manufacturer's instructions. A NanoDrop spectrophotometer (Thermo Scientific, Massachusetts, USA) was used for DNA quantification and quality checks, and the samples were stored at 4°C until sequencing.

### 2.4 16S rRNA Gene Sequencing and Data Analysis

The amplification of the *rrs* gene V4 region was performed via the primers 515F (GTGCCAGCMGCCGCGGTAA) and 806R (GGACTACNNGGGTATCTAAT) [12]. Sequencing was conducted by NOVOGENE Corporation (Beijing, China) through a NovaSeq 6000 (Illumina, USA), with a paired‐end length of 250 bp and a sequencing depth of 100k reads. The sequences were deposited in the NCBI Sequence Read Archive (SRA), Bioproject PRJNA1252344.

Bioinformatic analyses were conducted via Mothur v1.42.2 software [13]. All sequencing data without adapter sequences or barcodes were assembled into contigs; sequences without the expected length, more than eight homopolymers and ambiguities were removed, and identical sequences were merged. The sequences were then aligned via the SILVA v.132 [14] database as a reference, and the boundaries of the alignment were delimited. Sequences that did not align and unnecessary columns were removed. ASVs were clustered via the pre.cluster command and the Deblur algorithm [15]. The chimera.vsearch command was used to detect and remove chimeric sequences [16]. The sequences were classified via the Ribosomal Database Project (RDP) v.18 reference [17], and mitochondria, chloroplasts, archaea, eukaryotes, and unidentified sequences were removed. Singleton ASVs were excluded, and samples were randomly rarefied to the smallest size (27 720). The ASV distribution matrix, relative abundance profiles, and taxonomic classifications were exported from Mothur for statistical inference and plotting. Sample OL60.1 was removed because of low sequencing quality, and samples NAF15.3, NAF60.3, and MIX15.2 were identified as outliers and removed.

Past 4.6 software [18] was used to generate a principal coordinate analysis (PCoA) and a permutational multivariate analysis of variance (two‐way PERMANOVA) based on the ASV distribution matrix. An analysis of variance (ANOVA) or a Welch's *F* test (for homoscedastic and nonhomoscedastic data, respectively) was applied to the alpha diversity indices and relative abundance of taxa among the contaminants and times. Additionally, time and contaminants were considered independent factors for performing a two‐way ANOVA.

### 2.5 Functional Prediction of Hydrocarbon Degradation Potential

The metabolic potential of the microbial communities for hydrocarbon degradation was inferred via Phylogenetic Investigation of Communities by Reconstruction of Unobserved States (PICRUSt2; [19]). The predicted metagenomic functions were annotated against the Kyoto Encyclopedia of Genes and Genomes (KEGG) database, and KEGG Orthology (KO) identifiers related to hydrocarbon metabolism were extracted for downstream analyses.

## 3 Results

### 3.1 Bacterial Community Assessment

The microbial community richness (Sobs index) was altered by the different types of contaminants (two‐way ANOVA, *p* = 0.0001) but not by the different times (Figure 1a). Similarly, Shannon's index was statistically significant only for type (two‐way ANOVA, *p* < 0.0001) and not for time (Figure 1b).

**FIGURE 1:**
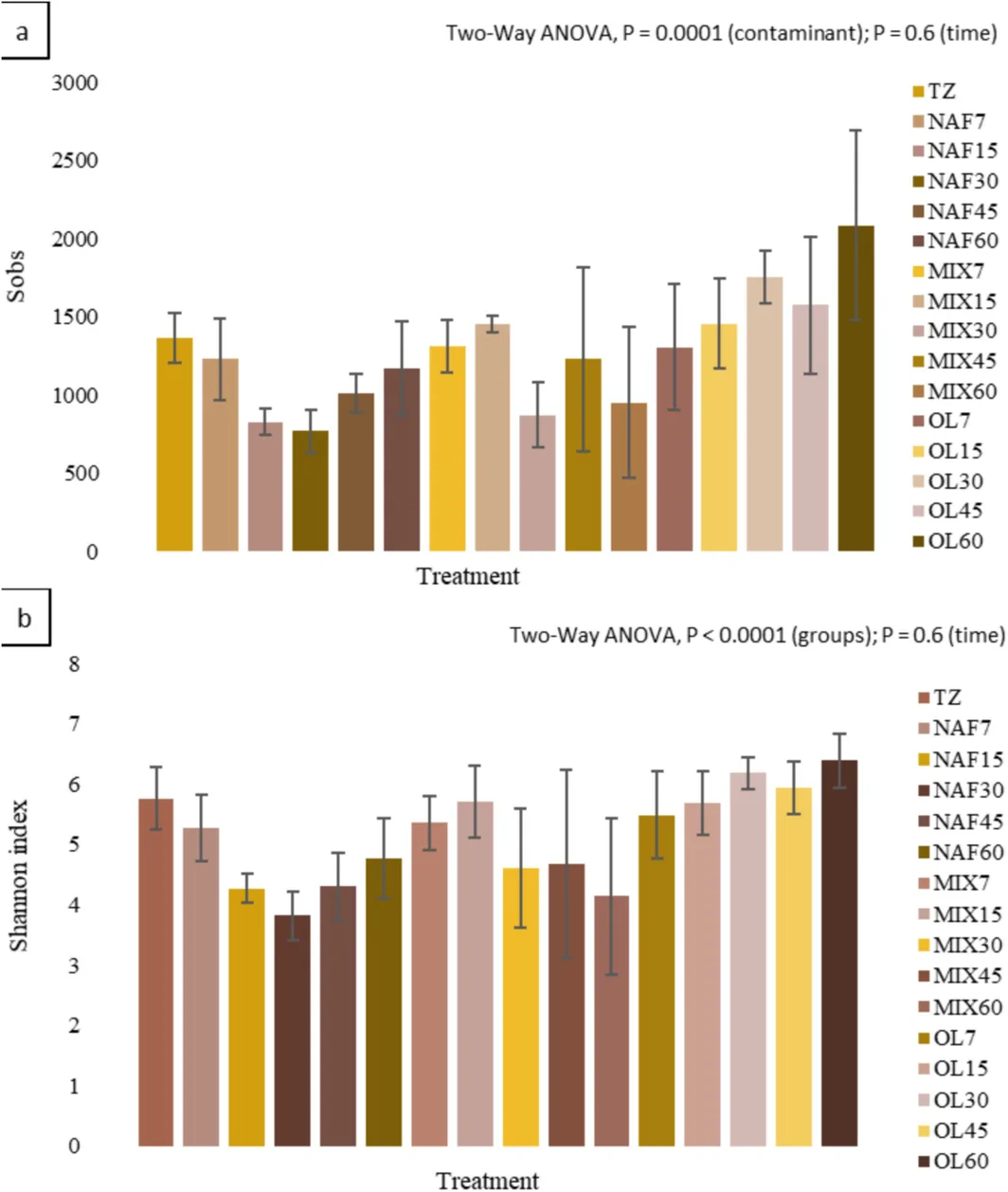
Mean richness and diversity of microbial communities, represented by the Sobs (a) and Shannon (b) indices, respectively, in marine water samples contaminated with different hydrocarbons—naphthalene (NAF), synthetic mixture (MIX), and crude oil (OL)—over various incubation times (7, 15, 30, 45, and 60 days). The control (TZ) contains only marine water and corresponds to time zero. The error bars represent the standard deviations. Two‐way ANOVA was used to test the effects of contaminant type and incubation time.

Principal coordinate analysis (PCoA) based on Bray–Curtis dissimilarity revealed clear differences in microbial community structure across treatments (Figure 2). The samples clustered distinctly according to the type of hydrocarbon contaminant (MIX, OL, or NAF), forming separate groups. The control group (TZ) also formed a distinct cluster, suggesting that the presence of hydrocarbons substantially altered the natural microbial community composition. These patterns were supported by two‐way PERMANOVA, confirming significant differences between contaminants (*p* < 0.0001) and between incubation times (*p* < 0.0001).

**FIGURE 2:**
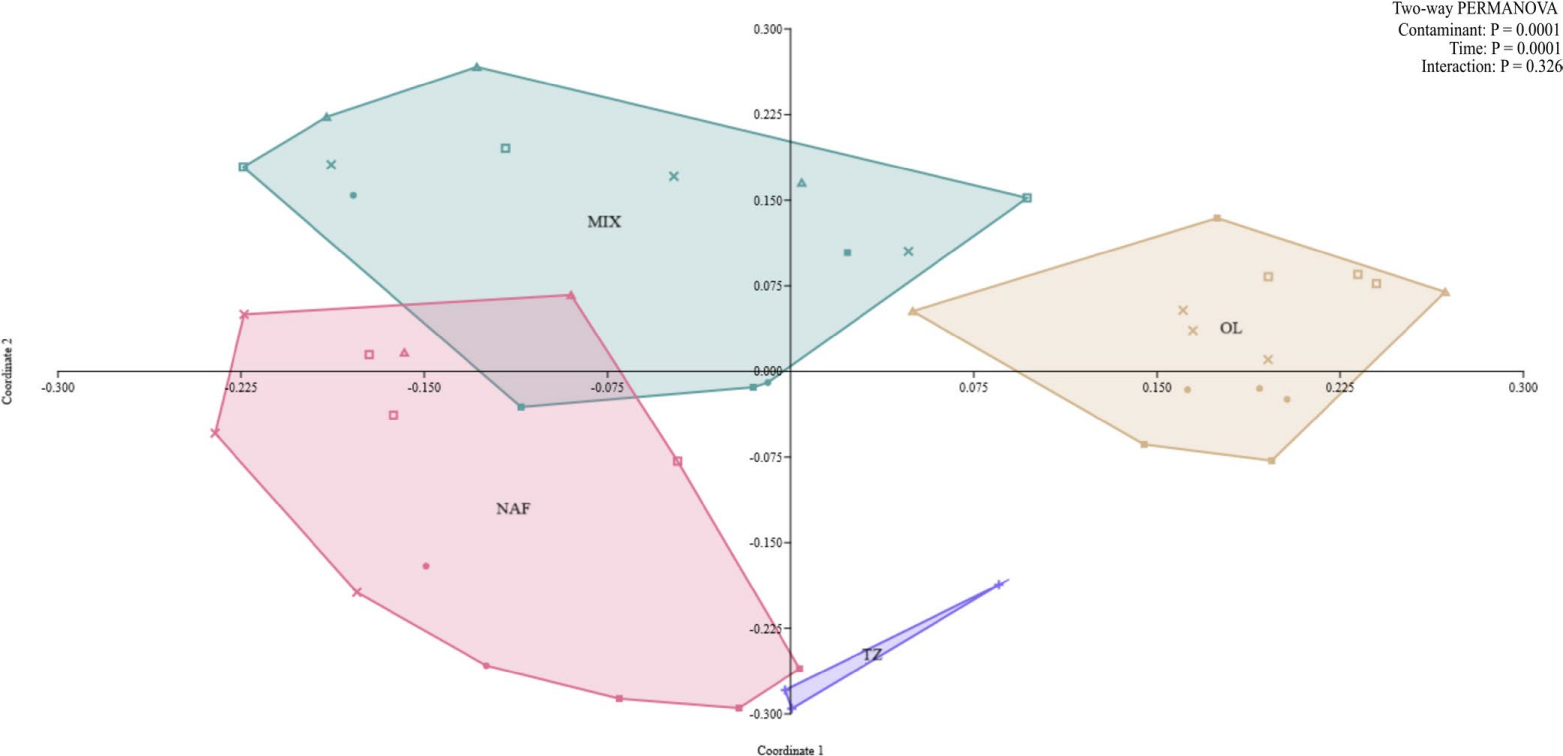
Principal coordinate analysis (PCoA) based on Bray–Curtis dissimilarity showing the microbial community structure across different hydrocarbon treatments—naphthalene (NAF), synthetic mixture (MIX), and crude oil (OL)—over multiple incubation times (7— filled square symbols, 15—dot symbols, 30—X symbols, 45— unfilled square symbols and 60—triangle symbols). The control (TZ) contains only marine water and corresponds to time zero (plus symbol). Two‐way PERMANOVA was applied to assess the effects of contaminant type, incubation time, and their interaction on community structure.

The relative abundance of the five main bacterial phyla, which represented 92% of the total, is shown in Figure 3. The most abundant phylum across all samples was Pseudomonadota, except for one replicate of OL7 (replicate 2) and one of OL60 (replicate 2), in which Bacillota was the most abundant. Other predominant phyla include Bacteroidota, Actinomycetota, and Acidobacteriota.

**FIGURE 3:**
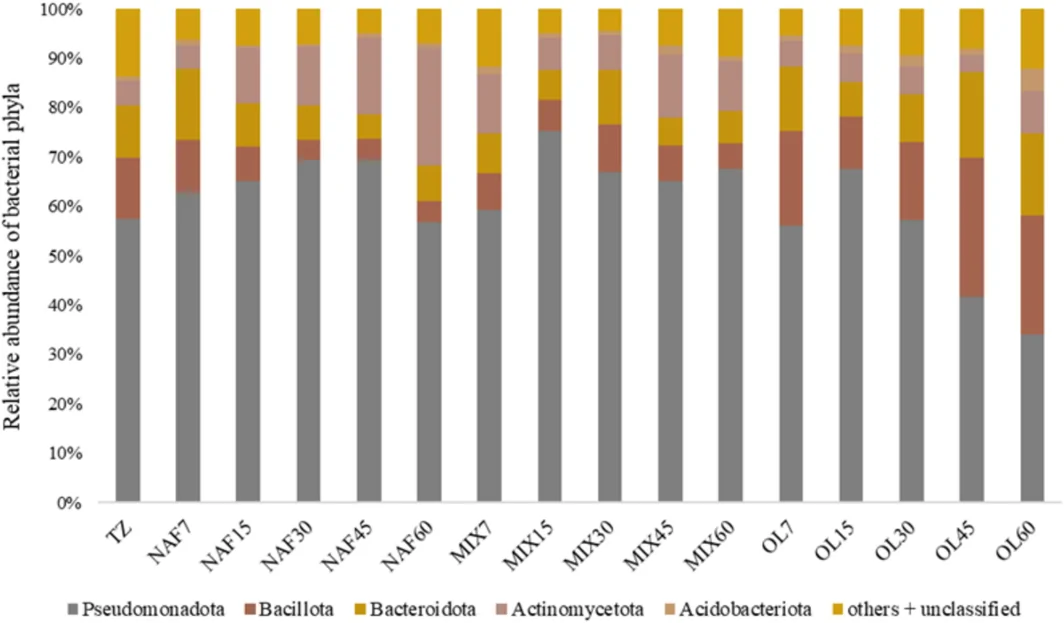
Relative abundance (average values) of bacterial phyla from marine water samples contaminated with different hydrocarbons—naphthalene (NAF), synthetic mixture (MIX), and crude oil (OL)—over various incubation times (7, 15, 30, 45, and 60 days). The control (TZ) contains only marine water and corresponds to time zero. A two‐way ANOVA was first applied to assess the overall effects of contaminant type, incubation time, and their interaction. Additionally, one‐way ANOVA or Welch's *F* test (for homoscedastic and nonhomoscedastic data, respectively) was used for group‐wise comparisons among individual treatment–time combinations.

Two‐way ANOVA revealed no significant differences over time for any phyla. However, significant differences between contaminants were found for the following groups: Bacillota was more abundant in OL than in MIX (*p* = 0.027); Acidobacteriota was more abundant in OL than in NAF (*p* = 0.019); and Actinomycetota was more abundant in NAF than in OL (*p* = 0.017). No significant interaction was detected for any phyla. ANOVA or Welch's *F* test (for homoscedastic and nonhomoscedastic data, respectively) was also performed for the five main phyla to compare contaminants and time points together. For this test, Acidobacteriota was the only phylum whose abundance significantly differed among the samples (*p* = 0.0004324). Tukey's post hoc test revealed differences between OL60 and NAF15 and between OL60 and NAF30 for this phylum.

The most abundant orders were Rhodospirillales, Clostridiales, Rhodobacterales, Alteromonadales, Bacteroidales, Pseudomonadales, and Oceanospirillales (Figure 4). Two‐way ANOVA revealed significant differences between contaminants for all orders, with the exception of Alteromonadales, which differed only between time points (*p* = 0.012). Rhodospirillales was different for both factors (contaminant—*p* = 0.0008; time—*p* = 0.049). No significant interaction was observed for any order.

**FIGURE 4:**
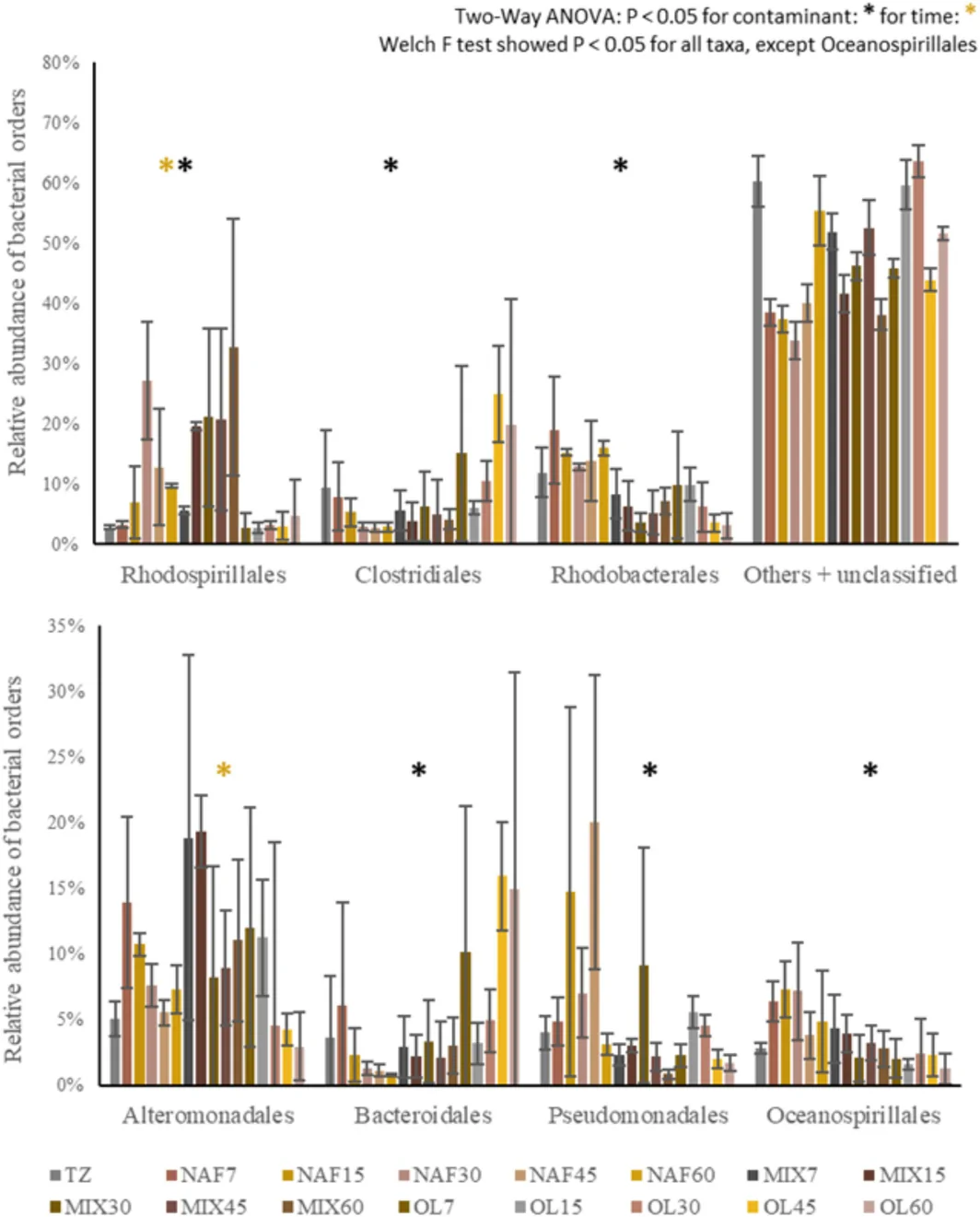
Relative abundance (average values ± SDs) of bacterial orders from marine water samples contaminated with different hydrocarbons—naphthalene (NAF), synthetic mixture (MIX), and crude oil (OL)—across various incubation times (7, 15, 30, 45, and 60 days). The control (TZ) contains only marine water and corresponds to time zero. A two‐way ANOVA was first applied to assess the overall effects of contaminant type, incubation time, and their interaction. Additionally, one‐way ANOVA or Welch's *F* test (for homoscedastic and nonhomoscedastic data, respectively) was used for group‐wise comparisons among individual treatment–time combinations.

Since the data were not homoscedastic, a Welch's *F* test was applied to compare contaminants and time points together, revealing differences for all orders except Oceanospirillales (*p* = 0.136). Tukey's post hoc test revealed differences between NAF45 and all the other samples, except MIX30, for Pseudomonadales. Rhodobacterales differed between NAF7 and MIX30, MIX45, MIX60, OL45, and OL60. A difference was also observed in Tukey's post hoc test between OL45 and both NAF30 and NAF45. Statistical analysis was not applied to “group + unclassified” since it included more than one taxon.

The 12 most abundant genera were *Thalassospira, Pseudomonas, Marinobacter, Alteromonas, Bacteroides, Muricauda, Hyphomonas, Duncaniella, Methylobacterium, Shimia, Marinobacterium*, and *Pseudidiomarina* (Figure 5).

**FIGURE 5:**
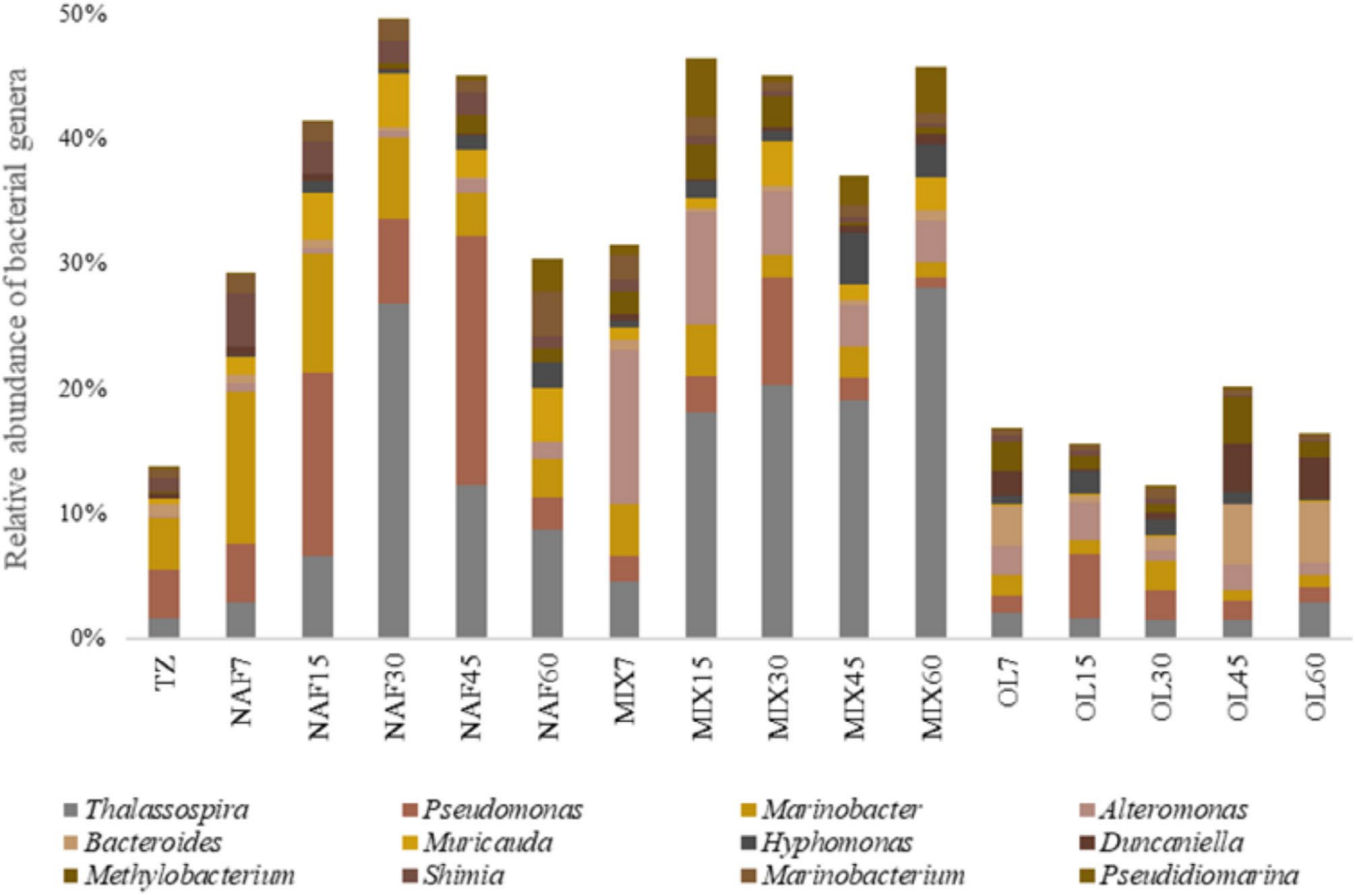
Relative abundance (average values) of the 12 dominant bacterial genera from marine water samples contaminated with different hydrocarbons—naphthalene (NAF), synthetic mixture (MIX), and crude oil (OL)—across various incubation times (7, 15, 30, 45, and 60 days). The control (TZ) contains only marine water and corresponds to time zero.

At time zero (TZ), *Marinobacter* was the most dominant genus (4.10%), followed by *Pseudomonas* (3.84%), *Thalassospira* (1.63%), *Shimia* (1.16%), and *Bacteroides* (1.08%). In the naphthalene treatment, on Day 7, *Marinobacter* remained predominant (12.19%), followed by *Pseudomonas* (4.77%), which became the most abundant genus on Day 15 (14.7%), *Marinobacter* (9.57%) and *Thalassospira* (6.6%). *Thalassospira* became dominant on Day 30 of incubation (26.83%), after which it was replaced again by *Pseudomonas* on Day 45 (19.9%). On the final day of incubation (Day 60), *Thalassospira* reached 8.63%, and other genera became slightly more prominent: *Muricauda* (4.29%) and *Marinobacterium* (3.65%). *Marinobacter* accounted for 3.08%, and *Pseudomonas* accounted for 2.68%.

In the MIX treatment, *Alteromonas* had the highest abundance on Day 7 (12.32%), whereas on the following days, this taxon decreased in abundance, and *Thalassospira* became predominant, increasing progressively over time, reaching 18.12% on Day 15 and attaining 28% on Day 60.

In the crude oil treatment, on Day 7, the relative abundance ranged from 1.39% to 3.28% for the following genera (in descending order): *Bacteroides, Alteromonas, Methylobacterium, Duncaniella, Thalassospira, Marinobacter*, and *Pseudomonas*. On subsequent days, although the dynamics of the most abundant genera changed, a greater diversity of genera compared with those in the NAF and MIX treatments remained consistently prominent throughout the 60‐day period, particularly the genera mentioned above.

The temporal variation in the six most abundant genera under the three contaminant conditions is shown in Figure 6. Overall, for all the taxa, crude oil presented the highest abundance, whereas naphthalene presented the lowest (Figure 6). This pattern was further supported by two‐way ANOVA, which revealed significant differences between contaminants for all taxa. Tukey's post hoc test results are presented in Table S1.

**FIGURE 6:**
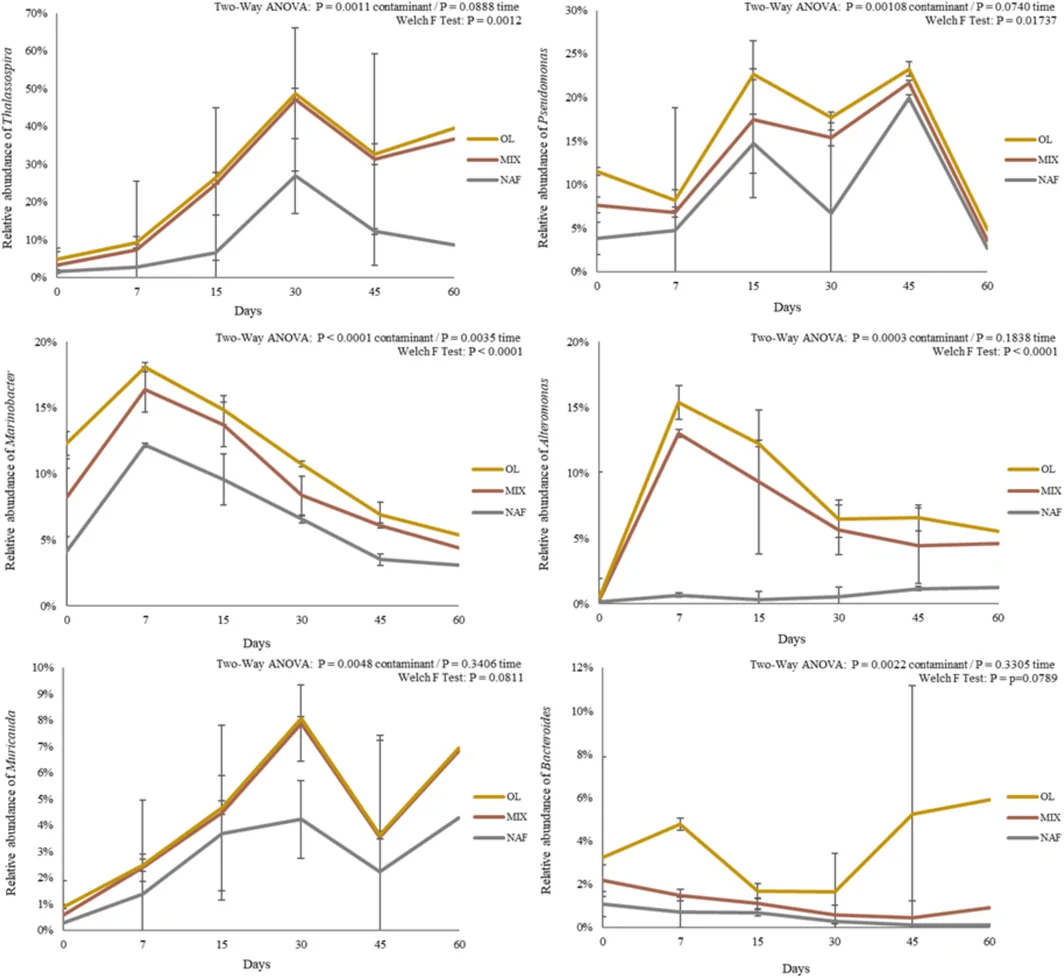
Relative abundance (average values ± SDs) of bacterial genera from marine water samples contaminated with different hydrocarbons—naphthalene (NAF), synthetic mixture (MIX), and crude oil (OL)—over time (7, 15, 30, 45, and 60 days). A two‐way ANOVA was first applied to assess the overall effects of contaminant type, incubation time, and their interaction. Additionally, one‐way ANOVA or Welch's *F* test (for homoscedastic and nonhomoscedastic data, respectively) was used for group‐wise comparisons among individual treatment–time combinations.

A similar temporal pattern was observed for the three contaminants (NAF, MIX, and OL), particularly *Thalassospira, Pseudomonas, Marinobacter*, and *Muricauda*. For *Thalassospira*, a peak in abundance was observed on Day 30 of incubation for all three contaminants. By Day 60, an increase was noted for OL and MIX, whereas a decrease occurred for NAF. For *Pseudomonas*, two peaks were detected—on Days 15 and 45—with a decline on Days 30 and 60 across all the treatments. *Marinobacter* sharply increased on Day 7, followed by a progressive decrease over time for all contaminants. For *Muricauda*, all three contaminants exhibited similar dynamics, with a steady increase leading to a peak on Day 30, followed by a decline on Day 45 and another increase on Day 60.

*Alteromonas* and *Bacteroides* exhibited distinct abundance patterns across the different contaminants. For *Alteromonas*, both OL and MIX peaked on Day 7, whereas NAF maintained low levels throughout the entire period. In contrast, *Bacteroides* was relatively stable under NAF and MIX conditions, whereas a peak was observed under OL conditions on Day 7, followed by a renewed increase beginning on Day 45.

Despite these visual differences, two‐way ANOVA indicated a statistically significant effect of time only for *Marinobacter*, specifically, between Day 7 and Day 45 (*p* = 0.0098) and between Day 7 and Day 60 (*p* = 0.0046). Welch's *F* test revealed significant differences for *Thalassospira* (*p* = 0.0012), *Pseudomonas* (*p* = 0.0173), *Marinobacter* (*p* < 0.05), and *Alteromonas* (*p* < 0.05), whereas *Muricauda* (*p* = 0.081) and *Bacteroides* (*p* = 0.078) showed marginal differences. A significant interaction was detected via two‐way ANOVA for *Marinobacter* and *Pseudomonas* (*p* = 0.049 for both).

Tukey's post hoc test revealed significant differences for *Alteromonas* between MIX7 and all time points for NAF and all time points for OL and TZ. For *Marinobacter*, a significant difference was observed between NAF7 and all the other samples, except NAF15 and NAF30; additionally, NAF15 differed from all the OL time points, as did MIX30, MIX45, and MIX60.

### 3.2 Predicted Hydrocarbon‐Degradation Functions (PICRUSt)

Four key functional markers identified in this study were selected to represent sequential stages of hydrocarbon degradation: K00496 (aliphatic oxidation; Figure 7a), K00529 (initial aromatic ring hydroxylation; Figure 7b), K00448 (ortho‐ring cleavage; Figure 7c), and K07104 (meta‐ring cleavage; Figure 7d). Although some other markers related to hydrocarbon‐associated metabolism were also detected, these were not further discussed, as they represent redundant (K00449—ortho ring cleavage, β subunit of protocatechuate 3,4‐dioxygenase), accessory (K01563—haloalkane dehalogenase involved in cometabolism and detoxification), or stress‐related (K04091—alkanesulfonate monooxygenase linked to sulfur scavenging and oxidative‐stress adaptation under nutrient limitation) functions rather than direct hydrocarbon catabolic pathways.

**FIGURE 7:**
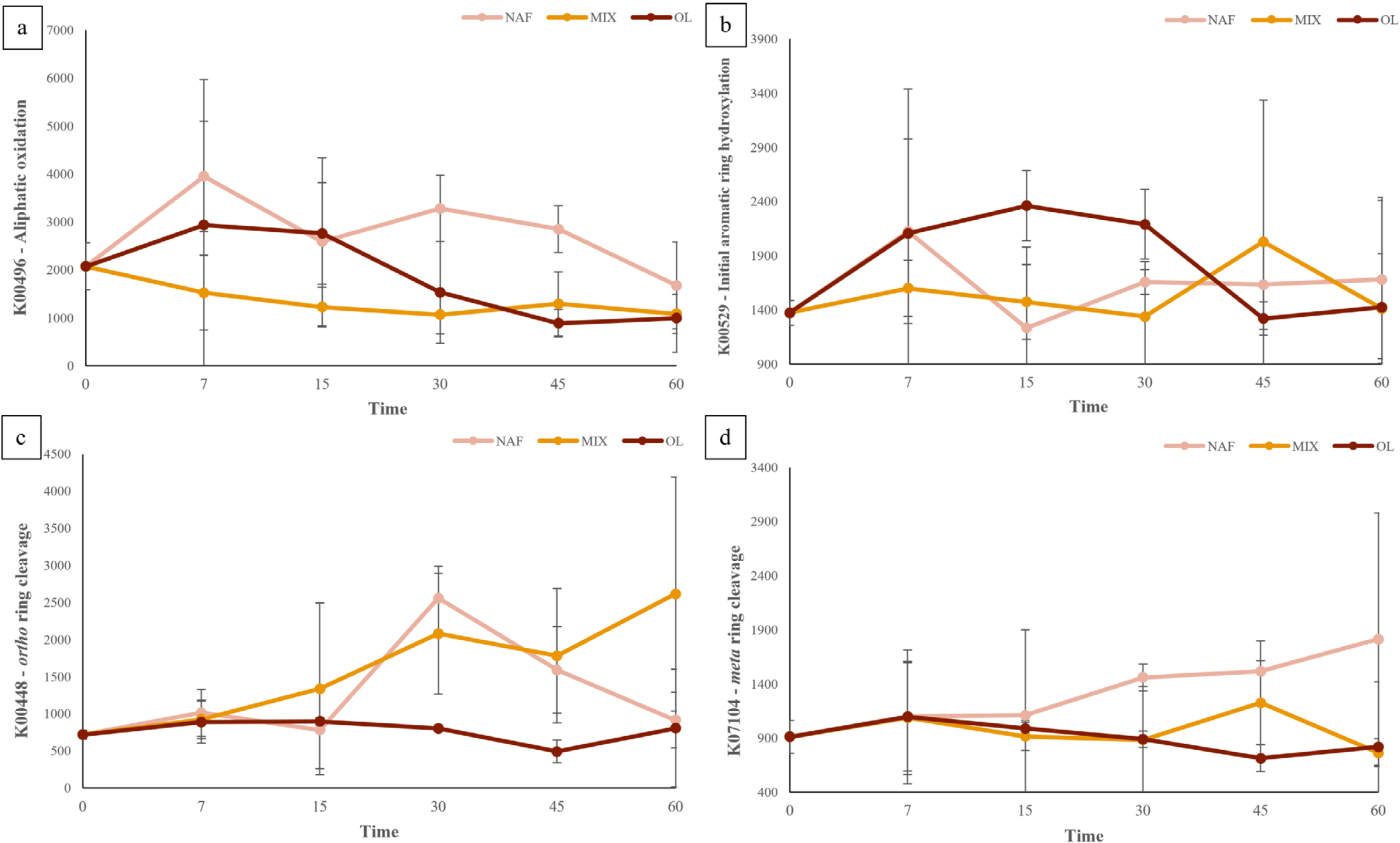
Predicted functional markers (average values ± SDs) associated with sequential stages of hydrocarbon degradation in marine microcosms contaminated with naphthalene (NAF), synthetic mixture (MIX), and crude oil (OL) over time (0—control, 7, 15, 30, 45, and 60 days). Functions correspond to (a) aliphatic oxidation (K00496), (b) initial aromatic ring hydroxylation (K00529), (c) ortho‐ring cleavage (K00448), and (d) meta‐ring cleavage (K07104). Predictions were obtained via PICRUSt2 on the basis of 16S rRNA gene profiles.

The alkane monooxygenase (alkB1_2, alkM—K00496), which is involved in the initial oxidation of short‐ and medium‐chain alkanes, showed early enrichment under NAF, peaking on Day 7 and again on Day 30, whereas the OL increased on Day 7 and remained relatively stable until Day 15 before declining. MIX gradually decreased over time, with a slight increase on Day 45.

The dioxygenase ferredoxin reductase component (hcaD—K00529), associated with the initial hydroxylation of aromatic rings, showed treatment‐specific responses. Under NAF, it peaked on Day 7 and decreased thereafter, with a smaller increase on Day 30. In OL, the values increased until Day 30 and declined afterwards, whereas in MIX, a modest increase was observed on Day 7, followed by a late peak on Day 45.

Protocatechuate 3,4‐dioxygenase (pcaG—K00448), a key enzyme of the ortho‐cleavage pathway, peaked on Day 30 under NAF and at both Days 30 and 60 under MIX, whereas OL remained stable throughout incubation, with a slight decrease on Day 45. Catechol 2,3‐dioxygenase (K07104), which is responsible for the meta‐cleavage of catecholic intermediates, showed a similar stable pattern in OL, whereas in NAF, it increased throughout the incubation period, especially from Day 30. During MIX, a transient increase was observed, especially on Day 45.

## 4 Discussion

Petroleum is a complex mixture of hydrocarbons that are biodegraded by diverse microbial communities, each with specific metabolic capabilities [20]. In this study, it was explored how different hydrocarbon types and exposure times influence community diversity, structure, and function, highlighting microbial succession patterns in marine water samples.

Richness and Shannon diversity differed significantly among contaminant types but not across timepoints. These findings suggest that hydrocarbon composition is a stronger driver of microbial community alpha diversity than temporal variation under the tested conditions. Complex mixtures, such as crude oil and synthetic mixture (MIX), appear to support more diverse communities likely related to the broader range of degradable substrates available, whereas single‐compound treatments such as naphthalene may promote the dominance of specific hydrocarbon‐degrading specialists, ultimately reducing overall community diversity [21, 22].

The distinct separation between the three contaminants (NAF, MIX, and OL) and the control (TZ) observed in the PCoA reinforces the strong influence of hydrocarbon type on shaping the microbial community structure. However, unlike the alpha diversity indices, time also influenced beta diversity, as observed via two‐way PERMANOVA and as previously reported [23, 24]. This suggests that shifts in community structure over time occurred independently of changes in alpha diversity.

An analysis of the composition of the bacterial communities revealed that the distribution of dominant bacterial phyla in response to different hydrocarbon contaminants clearly revealed patterns consistent with previously reported microbial dynamics in oil‐impacted environments: Pseudomonadota was the most abundant phylum across all the treatments, and other taxa, such as Bacillota, Bacteroidota, Actinomycetota, and Acidobacteriota, were also representative of contamination scenarios [20, 24, 25].

Thomas et al. [26] studied bacterial communities from marine water contaminated with oil and with oil + surfactants/emulsifiers over time. They also found genera from some orders identified as abundant in this study, such as Oceanospirilales, Pseudomonadales, and Alteromonadales. In addition, they observed succession in bacterial genera communities over time.

The relative abundance of bacterial genera across treatments and incubation times revealed clear shifts in community composition driven by hydrocarbon type. Some genera, such as *Thalassospira*, *Pseudomonas*, and *Marinobacter*, were expressed in all the contaminant types. *Alteromonas* was particularly abundant for MIX. These genera are well known for their hydrocarbon‐degrading potential and have been widely associated with the biodegradation of both aliphatic and aromatic compounds in marine environments [27, 28, 29].

A notable difference in bacterial succession dynamics was observed between the NAF and crude oil (OL) treatments. In the NAF, community shifts were characterized primarily by alternating dominance among three main genera: *Thalassospira*, *Marinobacter*, and *Pseudomonas*. In contrast, the crude oil treatment did not result in consistent dominance of any single genus. Instead, multiple genera, such as *Bacteroides*, *Alteromonas*, *Methylobacterium*, *Duncaniella*, *Thalassospira*, *Marinobacter*, and *Pseudomonas*, were maintained in varying proportions over time. This broader taxonomic diversity likely reflects the greater chemical complexity of crude oil, which can support a wider array of metabolic capabilities and is often associated with more complex microbial interactions reported in the literature [27, 30], as previously discussed.

The temporal dynamics of key bacterial genera across hydrocarbon treatments revealed distinct patterns of microbial succession shaped by both contaminant type and exposure time. *Marinobacter* and *Alteromonas* both presented relatively high abundances on Day 7, except for *Alteromonas* under NAF contamination, which declined steadily thereafter. Consistently, the functional profiles inferred by PICRUSt indicated early enrichment of alkane monooxygenase (K00496), particularly under NAF and OL, which may be linked to the dominance of these taxa. *Marinobacter* and *Alteromonas* are known to preferentially oxidize readily available substrates, initiating the early stages of aliphatic hydrocarbon degradation in marine environments [29, 31, 32].

The subsequent activation of the initial hydroxylation of aromatic rings (ring‐hydroxylating dioxygenase component K00529) is consistent with the onset of aromatic catabolism, which is typically initiated by primary aromatic degraders. *Pseudomonas* exhibited a biphasic response under OL and MIX, with distinct peaks at Days 15 and 45, which reflects its well‐documented metabolic versatility [28]—early activation linked to low‐molecular‐weight PAHs, followed by a secondary response associated with degradation intermediates and shifts in substrate availability. Recent studies have shown that *Pseudomonas* species can efficiently degrade simple aromatic compounds such as benzene and toluene through multiple dioxygenase pathways [33], suggesting that this genus plays a central role in the early stages of aromatic hydrocarbon degradation.

In contrast, the increase in protocatechuate 3,4‐dioxygenase (K00448), which is linked to the *ortho*‐cleavage pathway, was similar to the dominance of *Thalassospira*, particularly in the NAF and MIX treatments, consistent with reports of this taxon's involvement in the later stages of aromatic degradation. *Thalassospira* showed a progressive increase in relative abundance, peaking on Day 30 in all the treatments. This genus is known for its metabolic potential in complex contamination scenarios, supported by the presence of conserved pca genes (e.g., pcaG) encoding protocatechuate 3,4‐dioxygenase, which catalyzes downstream steps in the degradation of aromatic intermediates [34].

Finally, *Muricauda* increased gradually across treatments, with a peak at approximately Days 45–60, particularly in the OL. The abundance of *Bacteroides* displayed a unique trend in the OL treatments: an early increase, a moderate decrease, and a resurgence by Day 60. This pattern is consistent with enrichment in the late stages of degradation for both genera [35, 36]. This interpretation is consistent with the increase in catechol 2,3‐dioxygenase (K07104), a key enzyme of the *meta*‐cleavage pathway responsible for breaking down catecholic intermediates formed during aromatic degradation. This late activation of K07104 aligns with the rise of taxa involved in downstream transformations and carbon recycling and supports the idea that various community members may be enriched as part of complementary ecological functions rather than as primary degraders [6].

A key limitation of the present study is that microbial succession and functional potential were inferred from DNA‐based community profiles, without direct measurements of hydrocarbon degradation rates or intermediate metabolites. Consequently, increases in taxa previously associated with hydrocarbon degradation and functional predictions derived from bioinformatic approaches represent potential metabolic and ecological responses to hydrocarbon exposure rather than direct proof of functional activity and do not necessarily reflect gene expression or in situ biochemical processes.

Therefore, the patterns observed in this study reflect changes in microbial community structure and taxonomic succession, together with shifts in functional potential inferred from DNA‐based profiles, while interpretations linking these patterns to hydrocarbon degradation pathways are grounded in existing literature. Studies integrating chemical analyses with 16S rRNA‐based community profiling have shown that microbial succession during petroleum exposure closely follows temporal changes in hydrocarbon composition and substrate availability (e.g., [29, 37]).

More recent time‐resolved studies integrating chemical analyses with metagenomic approaches have shown that temporal shifts in microbial community structure are accompanied by changes in functional gene repertoires involved in hydrocarbon transformation, highlighting the value of combining chemical and high‐resolution molecular techniques [38]. These findings underscore the importance of future investigations coupling microbial community analyses with quantitative and activity‐based approaches.

## 5 Conclusion

This study demonstrated that hydrocarbon type is a major determinant of marine microbial community composition, with complex mixtures fostering greater diversity and more dynamic successional patterns than single‐compound treatments. Based on established literature on microbial hydrocarbon metabolism, functional predictions were consistent with the observed taxonomic succession, suggesting a potential shift in inferred metabolic capabilities from pathways associated with aliphatic hydrocarbon oxidation toward those related to aromatic compound transformation. These findings reinforce the importance of aligning bioremediation strategies with the specific chemical nature of the pollutants and the successional capacity of native microbial communities.

## Author Contributions

**Karen C. F. Santaren:** writing – review and editing, writing – original draft, methodology, formal analysis, data curation, conceptualization. **Douglas A. Monteiro:** writing – review and editing, methodology, investigation, formal analysis. **Eliene S. Lopes:** writing – review and editing, methodology, formal analysis. **Diogo A. Jurelevicius:** writing – review and editing, supervision, investigation, formal analysis, funding acquisition, conceptualization. **Lucy Seldin:** writing – review and editing, writing – original draft, supervision, resources, project administration, funding acquisition, conceptualization.

## Funding

This study was supported by grants from Conselho Nacional deDesenvolvimento Científico e Tecnológico (CNPq), Coordenação deAperfeiçoamento de Pessoal de Nível Superior (CAPES, financial code001) and Fundação de Amparo à Pesquisa do Estado do Rio de Janeiro(FAPERJ).

## Conflicts of Interest

The authors declare no conflicts of interest.

## Supporting information


**Table S1:** Tukey's post hoc test results for the six most abundant genera showing significant differences (*p* < 0.05 in red) between contaminant treatments (MIX—synthetic mixture, NAF—naphthalene, OL—crude oil).

## Data Availability

The data that support the findings of this study are available from the corresponding author upon reasonable request.
