# Supplementary material for: Microbial Community Dynamics in Marine Water: Influence of Hydrocarbon Type and Exposure Time
Source: Environ Toxicol. 2026 Mar 6;41(11):1265–76. doi: 10.1002/tox.70070 (PMC13656872; doi:10.1002/tox.70070)
Supplement: Supplementary file 1 [file TOX-41-1265-s001.docx]

**Microbial community dynamics in marine water: influence of hydrocarbon type and exposure time**

**Karen C.F. Santaren ^a^, Douglas A. Monteiro ^a^, Eliene S. Lopes ^a^, Diogo A. Jurelevicius ^a^, Lucy Seldin ^a*^**

^a^ Instituto de Microbiologia Paulo de Góes, Universidade Federal do Rio de Janeiro (UFRJ), Rio de Janeiro (RJ), Brazil.

**SUPPLEMENTARY MATERIAL**

| *Thalassospira* | NAF | MIX | OL |
| --- | --- | --- | --- |
| NAF |  | 0.2813 | 0.03415 |
| MIX | 0.2813 |  | 0.00061 |
| OL | 0.03415 | 0.00061 |  |
| *Pseudomonas* | NAF | MIX | OL |
| NAF |  | 0.005188 | 0.001739 |
| MIX | 0.005188 |  | 0.8986 |
| OL | 0.001739 | 0.8986 |  |
| *Marinobacter* | NAF | MIX | OL |
| NAF |  | 5.65E-05 | 1.06E-06 |
| MIX | 5.65E-05 |  | 0.2725 |
| OL | 1.06E-06 | 0.2725 |  |
| *Alteromonas* | NAF | MIX | OL |
| NAF |  | 0.00048 | 0.6636 |
| MIX | 0.00048 |  | 0.003778 |
| OL | 0.6636 | 0.003778 |  |
| *Bacteroides* | NAF | MIX | OL |
| NAF |  | 0.9746 | 0.006201 |
| MIX | 0.9746 |  | 0.009098 |
| OL | 0.006201 | 0.009098 |  |
| *Muricauda* | NAF | MIX | OL |
| NAF |  | 0.3675 | 0.003478 |
| MIX | 0.3675 |  | 0.07616 |
| OL | 0.003478 | 0.07616 |  |

**Table S1.** Tukey’s post hoc test results for the six most abundant genera. showing significant differences (P < 0.05. in red) between contaminant treatments (NAF – naphthalene. MIX – synthetic mixture. OL – crude oil).
